# Intake of ultra-processed foods is associated with inflammatory markers in Brazilian adolescents

**DOI:** 10.1017/S1368980021004523

**Published:** 2022-03

**Authors:** Glauciane Márcia dos Santos Martins, Ana Karina Teixeira da Cunha França, Poliana Cristina de Almeida Fonseca Viola, Carolina Abreu de Carvalho, Karla Danielle Silva Marques, Alcione Miranda dos Santos, Mônica Araújo Batalha, Janete Daniel de Alencar Alves, Cecilia Claudia Costa Ribeiro

**Affiliations:** 1 Federal University of Maranhão, Rua Barão de Itapary, University Hospital of the Federal University of Maranhão – HUUFMA, São Luís, MA, Brazil; 2 Federal University of Maranhão, Postgraduate Program in Collective Health, Rua Barão de Itapary, 155, Centro, São Luís, MA 65.020-070, Brazil; 3 Department of Nutrition, Federal University of Piauí, Teresina, PI, Brazil; 4 Federal University of Rio de Janeiro, Rio de Janeiro, RJ, Brazil; 5 Federal University of Maranhão, São Luís, MA, Brazil

**Keywords:** Food consumption, Ultra-processed foods, Processed foods, Inflammation, IL-8, Adolescents

## Abstract

**Objective:**

To evaluate the association of the consumption of foods of the ultra-processed group (UPF) with inflammatory markers in the adolescent population in Northeastern Brazil.

**Design:**

A cross-sectional population-based study. Food consumption was evaluated using two 24-h dietary recalls using the NOVA classification for food processing levels. The following inflammatory markers were evaluated: adiponectin, IL-6, IL-8, C-reactive protein (CRP) and TNF-*α*. Multivariate linear regression was used to investigate the association between the percentage of UPF energy contribution and inflammatory markers.

**Setting:**

São Luís, Maranhão, Brazil.

**Participants:**

The sample consisted of 391 male and female adolescents, aged from 17 to 18 years.

**Results:**

The average daily energy consumption by adolescents was 8032·9 kJ/d, of which 26·1 % originated from UPF. The upper tertile (T3) of UPF consumption presented higher intake of simple carbohydrates, lipids, saturated fat, and Na and lower protein intake. Individuals in T3 presented higher serum leptin and CRP levels (*P* < 0·05). Adolescents with UPF energy consumption ≥30·0 % (tertile 3 of UPF) had a 79 % (exp (0·58) = 1·79) increase in IL-8 levels when compared with adolescents in tertile 1 of UPF (*P* = 0·013).

**Conclusions:**

The association between the consumption of UPF, poor quality diet and pro-inflammatory markers have important harmful effects that can be observed as early as in adolescence.

The changes in global food consumption are related to diets high in fats, sugars and Na, which are chiefly linked to ultra-processed foods (UPF)^(1,2)^. In Brazil, the evolution of the household food availability from 2002–2003 to 2017–2018 showed a decrease in the participation of traditional foods such as rice, beans, cassava flour and milk and an increased participation of processed foods and industrialised mixtures in the Brazilian diet^(3)^. UPF, including breads, sausages, soft drinks, cookies and ready meals, account for 18·4 % of the Brazilian diet^(3)^. On the other hand, in the year 2000, the household consumption of UPF was recorded as over 50 % in middle- and high-income countries, like Chile, Canada and the United Kingdom^(4–6)^.

Adolescence is the period in life most prone to higher consumption of UPF. This factor leads to great biological and nutritional vulnerability of adolescents^(3,7)^. Therefore, it is relevant to investigate the effects of the early introduction of an unhealthy dietary pattern on the health of this population.

A higher intake of UPF has been directly associated with energy density, free sugars, saturated and *trans* fats levels, and inversely associated with the content of fibres, proteins, vitamins and minerals^(8,9)^. This poor dietary profile can induce postprandial metabolic changes^(10)^ and inflammation processes as it reduces antioxidant defences. It can also trigger oxidative stress and induce the transcription process of inflammatory genes, through the activation of the NF-*κ*B and the innate immune system^(11)^. Inflammatory responses are characterised by high synthesis and release of pro-inflammatory markers such as C-reactive protein (CRP), TNF-*α*, IL-6 and IL-8^(12)^, along with a decrease in the circulating levels of anti-inflammatory markers such as adiponectin^(13)^.

Studies have associated higher plasma concentrations of inflammatory markers and dietary patterns based on processed food and UPF or isolated nutrients (simple carbohydrates, saturated fat, *trans* fats and fibres)^(14,15)^. Such findings, however, are still limited and present conflicting results, especially in the young population. To date, no studies have investigated the relationship between UPF consumption and inflammatory markers in adolescents.

Because changes in inflammatory markers may be predictors of chronic non-communicable diseases, it is of great importance to study their associated factors such as food consumption. In this context, the objective of this study was to evaluate the association of UPF intake and inflammatory markers in adolescents from public schools in a capital city in the Northeastern region of Brazil.

## Methods

### Study design

The data used in this cross-sectional study originated from a research carried out with adolescents in the city of São Luís, state of Maranhão, between January 2014 and June 2016. The research entitled ‘Are oral disorders in adolescents associated with risk markers for chronic non-communicable diseases?’ investigated the association between nutritional and/or inflammatory markers and the outcomes of tooth decay, tooth loss, tooth infection and periodontal diseases in adolescents.

São Luís is the capital city of Maranhão, a state located in the Northeast of Brazil with a Human Development Index of 0·768^(16)^. In 2012, the urban area of São Luís had 42 009 high school students enrolled in fifty-two public schools.

### Study sample

The sample consisted of students enrolled in public high schools in São Luís – MA, Brazil. Initially, the high schools in the urban area were identified (*n* 52) and thirteen schools were randomly selected.

In the second phase, thirty-nine student classes were selected by chance from the previously selected schools (10, 11 and 12th grade). Students aged 17 or 18 years (*n* 2030) were considered eligible to enroll in the study.

The sample calculation considered a correlation coefficient between the percentage of UPF consumption and inflammatory markers of at least 15 %, power of the study at 80 % and 95 % CI, with a minimum sample size of 347 adolescents. A final sample of 391 adolescents had the UPF consumption assessed.

The Education State Department (SEDUC) provided a list with all public high schools in the urban area of São Luís. Afterwards, a three-stage cluster sampling (school, high school year and class) was carried out using the software Bioestat 5.3.

The sample was composed by students aged 17 or 18 years (*n* 2030) regularly enrolled in public schools, and the Free and Informed Consent Form was provided by their parents/guardians or on their own. Pregnant and lactating adolescents, individuals with low immunity associated diseases or under medications that could interfere with test results (corticosteroids or cytostatics) and those who did not respond to the dietary survey were not enrolled in this study.

### Data collection

Data were collected in the public schools in the city of São Luís by a multidisciplinary team composed of dentists, nutritionists and nutrition students.

A questionnaire on the socio-demographic and behavioural factors was applied to the adolescent and their guardian/parents. The following variables were analysed: sex (male and female); age (average ± sd); skin colour self-declaration using the classification of the Brazilian Institute of Geography and Statistics (IBGE) (white, black, yellow, brown and indigenous); maternal education (years of school attendance) (≤ 4; 5–8; 9–12 and > 12 years of school attendance); Brazilian Economic Classification in social classes (A/B, C and D/E) according to the Brazilian Association of Research Companies (ABEP)^(17)^; alcohol and tobacco consumption in the last year (yes and no); physical activity frequency using the Physical Activity Questionnaire for Adolescents (QAFA) (min/d)^(18)^.

### Nutritional assessment

The nutritional assessment was carried out by a group of trained nutritionists using anthropometric measurements and food consumption assessments. The anthropometric measurements (weight, height and waist circumference (WC)) were performed in duplicate considering the arithmetic mean value as final result. Weight was measured in a digital scale (Tanita®, Brazil), with maximum capacity of 150 kg and accuracy of 100 g. Participants were barefoot and wearing light clothing and were guided to stand in the centre of the scale. Height was measured using a portable stadiometer (Alturexata®), with 1·0 cm accuracy. The students stood in an upright position, barefoot, with their arms beside the body and with heels, back and back of the head making contact with the backboard.

WC was measured at the midpoint between the iliac crest and the last rib using a flexible and inelastic measuring tape (Sanny®). Body fat distribution was assessed by cut-off points proposed by Taylor *et al*.^(19)^. Abdominal fat was considered high when WC ≥ 80th percentile, adjusted for age and sex.

BMI was used to assess the ratio between weight and height ((kg)/height (m^2^)) and classified according to *Z*-score adjusted for sex and age. The recommendations by the WHO and used by the Ministry of Health of Brazil^(20)^ were as follows: underweight (<*Z*-score −2); eutrophy (≥*Z*-scores −2 and <*Z*-scores +1); overweight (≥*Z*-score- + 1 and <*Z*-score +2) and obesity (≥*Z*-score + 2).

Food consumption was assessed using two 24-h diet recall surveys. The first survey was applied on the same day as the anthropometric assessment and the second was applied a week later. The 24-h diet recall surveys were applied on non-consecutive days to minimise variation effects of intrapersonal food intake. Both recalls were considered in the analysis of average food consumption of the participating adolescents.

The 24-h diet recall was used to define and quantify food and beverage intake on the previous day, besides time, place, method of preparation and the brand of the reported items.

A photograph album with household utensils and food serving sizes extracted from the book ‘Photographic Record For Dietary Inquiry’^(21)^ was used to reduce the memory bias and assist in the identification of the reported servings.

Before recording the food consumption data, the collected information underwent quality control with the standardisation of food and beverage quantification, according to the Table for the Assessment of Household Food Consumption Measures^(22)^. Following, data were entered into the Virtual Nutri Plus® software and the information from the nutritional facts label that were not registered in the programme was included.

Foods were classified according to their level of processing, using the ‘NOVA classification’ with methodology proposed by Monteiro *et al*.^(23)^ and adapted by the Dietary Guidelines for the Brazilian Population^(2)^. Foods were classified into four groups: fresh or minimally processed foods; culinary ingredients; processed foods and UPF. In this study, we have classified the foods into three categories, merging the fresh or minimally processed foods group with the culinary ingredients group^(2,23)^.

The energy contribution percentage for each food group was calculated according to the level of processing, and the tertile of UPF contribution to the diet was assessed.

### Biochemical evaluation

Blood samples were used to analyse the following inflammatory markers: adiponectin, leptin, TNF-*α*, IL-6, IL-8 and high sensitivity CRP. The blood samples were collected by venipuncture in the morning after an overnight fast (12 h) by a nurse in the schools. The samples were identified, stored in a styrofoam box with ice pads, transported to laboratory and processed on the same day. Inflammatory markers were determined using the Magpix-Milliplex technology.

The high sensitivity CRP was classified according to Pearson *et al*.^(24)^; values above 3·0 mg/l and below or equal to 10·0 mg/l were considered inflammation; values above 10·0 mg/l were considered acute inflammatory processes. Other inflammatory markers had no reference values.

### Statistical analysis

Categorical variables were presented as frequencies and percentages and all quantitative variables were presented as means and standard deviations (± sd). Variable normality was evaluated by the Kolmogorov–Smirnov test.

Energy and nutrient profiles were analysed according to tertiles of the UPF contribution using ANOVA with Bonferroni correction (Bonferroni post hoc test). The Pearson’s *χ* ^2^ test was used to compare socio-demographic, body composition and behaviour with the tertile of the UPF contribution.

A linear regression model was used to assess the association between inflammatory markers adiponectin, leptin, IL-6, IL-8, TNF*α* and CRP (dependent variable) and tertiles of UPF intake (main independent variable). For each inflammatory marker, a crude and adjusted model was constructed. A univariate linear regression model was initially carried out between inflammatory markers and tertiles of UPF consumption.

The models were adjusted for potential confounding factors (independent variables), such as demographic (sex), socio-economic (skin colour, economic classification and maternal education), anthropometric measures (BMI and WC) and behavioural factors (smoking and alcohol consumption). Variables with non-normal distribution were log transformed. Statistical analyses were performed using the STATA® Program (version 14.0), and the statistical significance level used for all analyses was 5 %.

## Results

The study included 391 adolescents with a prevalence of female students (57·0 %) and age mean of 17·3 (± 0·49) years. The study showed a higher prevalence of participants self-declared as brown (64·9 %), maternal education between 9 and 12 years (40·4 %), belonging to Economic Class C (64·6 %). Most adolescents were insufficiently physically active (51·2 %) and reported not having consumed tobacco (87·7 %) or alcohol (53·2 %) in the last year. WC was within normal range (79·5 %), and BMI showed that 18·7 % were overweight or obese. The comparison between socio-demographic, body composition and behavioural factors and the UPF energy contribution tertiles showed an association between adolescents who consumed tobacco and the lowest tertile (T1) of ultra-processed consumption (*P* < 0·05) (Table 1).

**Table 1:** Association of socio-demographic, anthropometric and behavioural characteristics with tertiles of ultra-processed food contribution to the diet of adolescents. São Luís – MA, Brazil

	Ultra-processed food consumption								
	Total		T1		T2		T3		
Variable	%	*n*	%	*n*	%	*n*	%	*n*	P*
Sex									
Male	43	168	35·1	59	35·1	59	29·8	50	0·446
Female	57	223	32·3	72	31·8	71	35·9	80	
Skin colour									
White	15·5	60	33·3	20	40·0	24	26·7	16	0·442
Black	18·7	72	26·4	19	36·1	26	37·5	27	
Brown	65·8	254	35·4	90	31·5	80	33·1	84	
Maternal schooling									
≤ 4 years	20·9	71	33·8	24	33·8	24	32·4	23	0·398
5–8 years	26·0	88	39·8	35	29·6	26	30·7	27	
≥9 years	53·1	180	27·8	50	36·7	66	35·6	64	
Brazilian economic classification									
A/B	15·5	60	26·7	16	36·7	22	36·7	22	0·230
C	64·6	250	31·6	79	34·4	86	34·0	85	
D/E	19·9	77	44·2	34	28·6	22	27·3	21	
Physical activity									
Sufficiently active	48·8	191	35·6	68	30·9	59	33·5	64	0·570
Insufficiently active	51·2	200	31·5	63	35·5	71	33·0	66	
Alcohol consumption in the past year									
Yes	46·3	179	36·9	66	34·1	61	29·1	52	0·234
No	53·7	208	30·3	63	33·2	69	36·5	76	
Tobacco consumption in the past year									
Yes	11·4	44	50·0	22	29·6	13	20·5	9	0·034
No	88·6	343	31·2	107	34·1	117	34·7	119	
BMI classification									
Underweight/eutrophy	81·3	317	34·1	108	33·4	106	32·5	103	0·762
Overweight/obesity	18·7	73	31·5	23	31·5	23	37·0	27	
Waist circumference									
Normal	82·7	311	34·4	107	35·4	110	30·2	94	0·129
High	17·3	65	29·2	19	27·7	18	43·1	28	

* Pearson’s χ^2^ test.

**Fig. 1:**
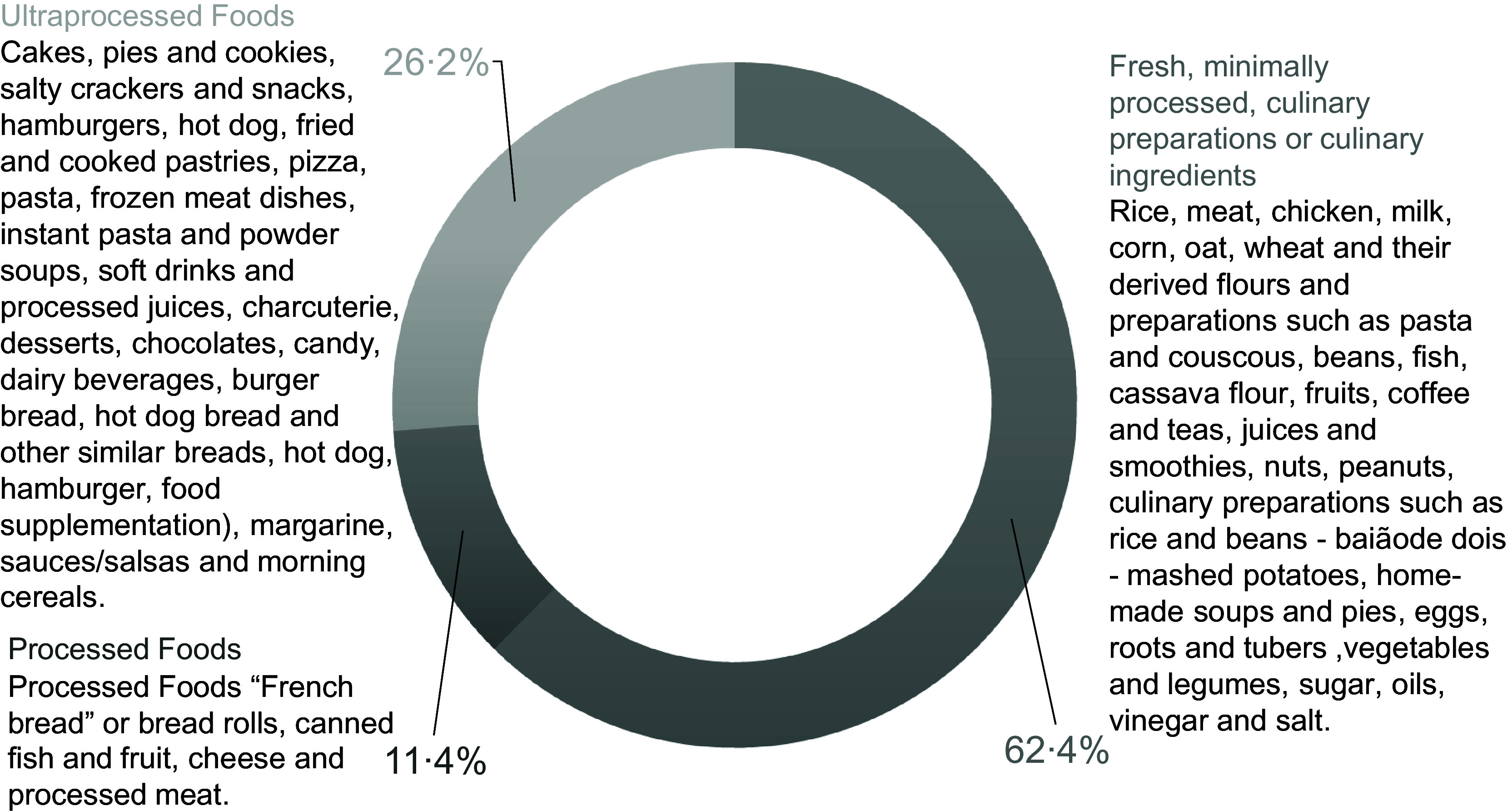
Consumption of fresh foods, minimally processed foods, culinary preparations, culinary ingredients, processed and ultra-processed foods of adolescents, São Luís – MA, Brazil

The average energy consumption for the adolescent group was 8032·9 kJ/d (1919·0 kcal/d), of this 62·4 % derived from fresh food, minimally processed foods, processed culinary ingredients or culinary preparations; 11·4 % from processed foods and 26·2 % from UPF (Fig. 1). The foods with the highest energy contributions among fresh foods, minimally processed foods and culinary preparations were rice (14·8 %), meat (10·4 %), chicken (7·3 %) and milk (4·9 %). Those with the lowest contributions were roots and tubers (0·6 %) and vegetables (0·3 %). In the UPF group, the items with the highest energy participation were white bread (10·2 %) and canned fish (0·6 %). Among the UPF, cakes, pies and sweet cookies (5·6 %), crackers and chips (4·4 %) and fast food (3·6 %) stood out in the total daily energy consumed by adolescents (Table 2).

**Table 2:** Averages of absolute and relative consumption of fresh foods, minimally processed foods, culinary preparations, culinary ingredients, processed and ultra-processed foods of adolescents, São Luís – MA, Brazil

Variable	kJ/d	kcal/d	%total energy intake
Fresh, minimally processed, culinary preparations or culinary ingredients	5012·7	1197·5	62·4
Rice	1164·1	278·1	14·8
Meat	839·7	200·6	10·4
Chicken	572·2	136·7	7·3
Milk	407·3	97·3	4·9
Other cereals and preparations*	285·5	68·2	3·5
Beans	232·3	55·5	2·9
Fish	232·3	55·5	3·0
Cassava flour	220·2	52·6	2·5
Fruits	203·4	48·6	2·4
Coffee and teas	142·7	34·1	1·9
Juices and smoothies	159·7	36·0	‘1·8
Other food and mixed preparations^†^	141·1	33·7	1·8
Eggs	126·4	30·2	1·6
Roots and tubers	47·3	11·3	0·6
Vegetables and legumes	20·9	5·0	0·3
Sugar	217·2	51·9	2·6
Oils	9·2	2·2	0·1
Others^‡^	0·0	0·0	0·0
Processed foods	895·0	213·8	11·4
Bread^§^	815·9	194·9	10·2
Canned fish and fruit	34·3	8·2	0·6
Cheese	33·5	8·0	0·4
Processed meat	11·3	2·7	0·2
Ultra-processed food	2122·2	507·7	26·2
Cakes, pies and cookies	477·2	114·0	5·6
Salty crackers and snacks	360·7	86·7	4·4
Fast food meals^‖^	239·1	57·2	3·6
Ready and semi-ready meals^¶^	224·8	53·7	2·8
Soft drinks and processed juices	206·9	49·5	2·4
Charcuterie	164·7	39·4	2·1
Desserts, chocolates, candy	142·5	34·1	1·8
Dairy beverages	129·3	30·9	1·6
White bread**, hot dog, hamburger	128·1	30·6	1·5
Other products^††^	23·9	5·7	0·2
Margarine and sauces/salsas	21·3	5·1	0·2
Morning cereals	3·3	0·8	0·0
Total	8032·9	1919·0	100·0

* Includes maize, oat, wheat and their derived flours and preparations such as pasta and couscous.

† Includes nuts, peanuts, culinary preparations such as rice and beans (baião de dois), mashed potatoes, home-made soups and pies.

‡ Includes vinegar and salt.

§ Includes ‘French bread’ (bread rolls).

‖ Includes hamburgers, hot dog, fried and cooked pastries.

¶ Includes pizza, pasta, frozen meat dishes, instant pasta and powder soups.

** Includes industrialised breads: burger bread, hot dog bread and other similar breads.

†† Includes food supplementation.

The consumption of UPF ranged from an average of 6·0 % of the total energy in tertile1 (T1) to 48·2 % in tertile 3 (T3). The analysis of the contribution of total energy and nutrients consumed in these tertiles show that there was a higher intake of simple carbohydrates, lipids, saturated fat and Na in T2 and T3 compared with T1 (*P* < 0·05). On the other hand, there was a higher protein intake in T1 and T2 compared with T3 (*P* < 0·05) (Table 3).

**Table 3:** Tertiles of ultra-processed food contribution to the total energetic and nutritional intake by adolescents. São Luís – MA, Brazil

	T1		T2		T3		
	(≤15·9 %)		(>15·9 e < 30 %)		(≥30·0 %)		
Energy/nutrients	Mean	sd	Mean	sd	Mean	sd	P^*^
Energy (kJ/d)							0·342
(kcal/d)	1860·5	683·1	1981·6	657·8	1914·2	663·8	0·342
Carbohydrate (g)	140·6	24·0	146·0	24·1	147·1	25·6	0·073
Simple carbohydrate (kcal/d)	217·8^†^,^‡^	157·9	301·0	166·2	342·7	183·4	< 0·001
Protein (g)	52·7^†^,^‡^	15·1	46·6^§^	10·7	42·1	9·6	< 0·001
Lipids (g)	31·2^†^	6·3	32·9^§^	6·0	36·1	6·7	< 0·001
Saturated fat (g)	9·8^†^,^‡^	3·0	10·8	3·5	11·4	3·5	< 0·001
Polyunsaturated fat (g)	4·5	2·1	4·5	2·0	5·0	2·7	0·087
Monounsaturated fat (g)	8·1	3·0	8·2	3·2	8·0	3·2	0·863
Fibre (g)	8·5^‡^	4·3	8·3	4·7	7·2	3·4	0·025
Vitamin A (mg)	525·8	1442·9	426·7	922·1	480·2	1360·7	0·818
Vitamin B_6_ (mg)	0·6	0·3	0·6	0·3	0·5	0·3	0·188
Vitamin C (mg)	56·4	134·9	59·4	101·2	47·0	120·1	0·684
Vitamin D (mcg)	1·7	1·4	1·5	0·9	1·4	1·0	0·040
Vitamin E (mg)	5·9	3·1	6·1	3·4	6·8	4·3	0·111
Ca (mg)	259·6	90·0	251·8	104·7	246·8	111·1	0·596
Na (mg)	1275·4^‡^	418·4	1370·8	432·9	1478·6	518·8	0·002

Todos os nutrients em g/mg estão ajustados por 1000 kcal (4186 kJ);

* ANOVA test, com teste pós hoc de Bonferroni.

† *P* value < 0·05 T1 *v*. T2.

‡ *P* value < 0·05 T1 *v*. T3.

§ *P* value < 0·05 T2 *v*. T3.

Mean values of inflammatory markers were: 0·041 mg/l for adiponectin, 0·051 mg/l for lepin, 1·18 mg/l for IL-6, 27·13 mg/l for IL-8, 0·11 mg/l for CRP and 2·11 mg/l for TNF-*α*.

Results from the adjusted analysis of the linear regression model are shown in Table 4. Adolescents with UPF energy consumption ≥30·0 % (tertile 3 of UPF) had a 79 % (exp (0·58) = 1·79) increase in IL-8 levels when compared with adolescents in tertile 1 of UPF.

**Table 4:** Association between the tertiles of the percentage of energetic contribution of ultra-processed foods (UPF) to the diet of adolescents and inflammatory markers. São Luís – MA, Brazil

Inflammatory marker	Non-adjusted model			Adjusted model*		
	*β*	95 % CI	*P*	*β*	95 % CI	*P*
Model 1 adiponectin						
Tertile 1 of UPF (≤15·9 %)	Ref			Ref		
Tertile 2 of UPF (>15·9 e < 30 %)	−0·02	−0·18, 0·14	0·800	0·00	−0·18, 0·19	0·972
Tertile 3 of UPF (≥30·0 %)	−0·09	−0·26, 0·07	0·252	−0·10	−0·28, 0·08	0·272
Model 2 leptin						
Tertile 1 of UPF (≤15·9 %)	Ref			Ref		
Tertile 2 of UPF (>15·9 e < 30 %)	0·06	−0·35, 0·47	0·772	0·04	−0·24, 0·33	0·753
Tertile 3 of UPF (≥30·0 %)	0·38	−0·02, 0·79	0·065	0·38	−0·10, 0·45	0·219
Model 3 IL-6						
Tertile 1 of UPF (≤15·9 %)	Ref			Ref		
Tertile 2 of UPF (>15·9 e < 30 %)	0·07	−0·16, 0·30	0·564	0·17	−0·08, 0·41	0·179
Tertile 3 of UPF (≥30·0 %)	0·09	−0·14, 0·31	0·449	0·16	−0·08, 0·40	0·197
Model 4 IL-8						
Tertile 1 of UPF (≤15·9 %)	Ref			Ref		
Tertile 2 of UPF (>15·9 e < 30 %)	0·24	−0·17, 0·65	0·247	0·31	−0·15, 0·77	0·186
Tertile 3 of UPF (≥30·0 %)	0·39	−0·22, 0·80	0·064	0·58	0·12, 1·00	0·013
Model 5 TNF-*α*						
Tertile 1 of UPF (≤15·9 %)	Ref			Ref		
Tertile 2 of UPF (>15·9 e < 30 %)	−0·10	−0·29, 0·10	0·330	−0·02	−0·23, 0·19	0·854
Tertile 3 of UPF (≥30·0 %)	−0·02	−0·22, 0·17	0·818	0·00	−0·21, 0·21	0·999
Model 6 C-reactive protein						
Tertile 1 of UPF (≤15·9 %)	Ref			Ref		
Tertile 2 of UPF (>15·9 e < 30 %)	0·01	−0·34, 0·36	0·964	−0·05	−0·43, 0·33	0·812
Tertile 3 of UPF (≥30·0 %)	0·30	−0·05, 0·65	0·095	0·26	−0·12, 0·64	0·182

* Model adjusted for sex, skin colour, maternal schooling, economic class, physical activity, alcohol consumption, tobacco consumption, BMI and waist circumference.

## Discussion

In this study carried out in adolescents, over 25 % of energy intake originated from UPF (26·2 %). Subjects of this food group have especially high consumption of simple carbohydrates, lipids, saturated fat and Na, and lower intake of proteins and fibre. The regression model adjusted for the variables (sex, skin colour, maternal education and economic class, physical activity, alcohol and tobacco consumption, BMI and WC) confirmed the association between higher UPF consumption and higher IL-8 levels.

The use of the cross-sectional design in the present study was considered a limitation; thus, the effect of UPF consumption on the occurrence of chronic outcomes may be better determined in a longitudinal study. However, the effect of a diet rich in UPF on metabolic and inflammatory alterations has also been investigated in a short-term randomised clinical trial^(25)^.

In addition, the external validity of the results may have also been limited by the participation of students exclusively from public schools. It is possible that this factor could have underestimated the observed associations because students from private schools, with higher socio-economic status, tend to show a higher consumption of UPF and, hence, the impact on the inflammatory markers may also be greater^(3)^. Nonetheless, we have found relevant results, which demonstrate that adolescents in the groups with the highest UPF intake may have significant alterations of their inflammatory state.

On the other hand, the strengths of this study include its innovative character, the probability sampling, the assessment of food consumption using the NOVA classification and the use of early inflammatory markers in a young population that may limit reverse causality, which is a common limitation of studying the association between UPF and chronic disease health outcomes cross-sectionally. Another strong point was the investigative method for the study of food consumption, the 24-h diet recall, which despite its limitations, is able to quantify all the food consumed by the interviewees. The questionnaire was applied in two moments, increasing its ability to describe diet habits of the subjects.

The percentage of UPF energy contribution observed in our sample group was lower than that observed in other studies with adolescents in Brazil^(26)^. A study carried out in 2016 with 2499 adolescents in the same age group and city of the present study found that 35·8 % of the total energetic intake originated from UPF^(27)^.

The lower prevalence of UPF consumption found in the present study can be associated with the difference in the socio-economic levels of the participants. In this work, the sample was composed of adolescents, mostly belonging to Class C from public schools in a city of the Northeast region in Brazil, which is the poorest region of the country. Sousa *et al*.^(27)^ carried out a study in the same city, but participants belonged mostly to Class B. Other studies conducted in the Southern region of Brazil also included private schools with better socio-economic conditions. It must be noted that in Brazil, lower socio-economic status is correlated with a lower consumption of UPF^(3)^.

This study identified that as the energy participation of UPF increases, there is a decrease in the quality of the diet due to a higher intake of simple carbohydrates, lipids and saturated fat, and lower protein intake. Louzada *et al.* ^(9)^ observed higher consumption of sugars, total, saturated and *trans* fat, and lower consumption of proteins, fibres and K in the highest quintile of this group. Similar results have been reported in other studies carried out in the USA, Colombia, the United Kingdom and Canada^(28–31)^.

In agreement with other studies, for example, those by D’Avila *et al.* ^(26)^ and Melo *et al*.^(32)^, we found no association between the consumption of UPF and overweight/obesity and abdominal fat in adolescents. Contrarily, two other studies that assessed the data from the 2008–2009 POF (Consumer Expenditure Survey) contradicted our results. A study carried out with 30 243 adolescents/adults found that those in the highest quintile of UPF intake had significantly higher BMI, with higher probability of overweight and obesity^(33)^. Another work assessed 55 970 Brazilian families and found that the domestic availability of UPF was also positively associated with BMI and the prevalence of overweight and obesity^(34)^. Monteiro *et al*.^(35)^ identified the same positive association in nineteen European countries.

It is of note that both the above-mentioned studies with data from POF (Consumer Expenditure Survey) had a more robust probability sampling and broader age groups. Thus, although this study with adolescents from public schools showed no association between UPF intake and overweight/obesity and abdominal fat, it is important to keep in mind that these individuals may express harmful effects of this food consumption pattern in future stages of life.

Studies assessing the association of UPF intake with inflammatory markers are still scarce in the literature. So far, the only study on the association between UPF intake and the inflammatory marker CRP was carried out with adults from the ELSA cohort (Brazilian Longitudinal Study of Adult Health)^(36)^. Their results revealed that, among women, this association was not significant after BMI adjustment, which points to a possible mediation effect by adiposity^(36)^.

This is the first study on the relationship between inflammatory markers (adiponectin, leptin, IL-6, IL-8, CRP and TNF-*α*) and the consumption of UPF among adolescents using the NOVA classification. Individuals with the highest UPF energy contribution (T3) had increased serum levels of leptin and CRP when compared to the individuals with the lowest contribution (T1), in addition to a higher level of IL-8 even after adjusting for confounding variables.

We suggest that this result can be explained by the ability of certain nutrients to modulate the inflammatory response. The increased intake of simple carbohydrates and saturated fats (widely present in UPF) can lead to the excessive production of reactive oxygen species and promote chronic inflammation with the increase of CRP, leptin, IL-6 and TNF-*α*, and the reduction of adiponectin^(37–39)^. These markers can control the interactions between immune system cells and regulate local and systemic inflammatory responses^(40)^. The inadequate control of the inflammatory process may evolve into a low-grade chronic inflammation^(41)^ with harmful effects such as tissue damage due to a prolonged activation of the innate immune system. This could lead to the occurrence and progression of several non-communicable diseases such as CVD, obesity, diabetes mellitus and certain types of cancer^(42)^. The relationship between inflammatory markers and eating habits in adolescence has been confirmed and assessed by other methods that did not consider the food processing levels^(14,43,44)^.

Thus, the association of UPF consumption with inflammatory markers points to yet another harmful effect resulting from the high consumption of this food group in younger individuals. It indicates that the early exposure to dietary risk factors leads to health consequences and may cause more severe problems in the long term, since the triggering of chronic low-grade inflammation is an important risk factor for the occurrence of non-communicable diseases.
